# Disease activity and erectile dysfunction in Japanese patients with ulcerative colitis

**DOI:** 10.1093/sexmed/qfad024

**Published:** 2023-05-26

**Authors:** Shinya Furukawa, Eiji Takeshita, Teruki Miyake, Kazuhiro Tange, Hideomi Tomida, Yasunori Yamamoto, Yoshio Ikeda, Yoichi Hiasa

**Affiliations:** Health Services Center, Ehime University, Matsuyama, Ehime 790-8577, Japan; Department of Inflammatory Bowel Diseases and Therapeutics, Ehime University Graduate School of Medicine, Shitsukawa, Toon, Ehime 790-0295, Japan; Department of Gastroenterology and Metabology, Ehime University Graduate School of Medicine, Shitsukawa, Toon, Ehime 790-0295, Japan; Department of Inflammatory Bowel Diseases and Therapeutics, Ehime University Graduate School of Medicine, Shitsukawa, Toon, Ehime 790-0295, Japan; Endoscopy Center, Ehime University Hospital, Shitsukawa, Toon, Ehime 791-0295, Japan; Endoscopy Center, Ehime University Hospital, Shitsukawa, Toon, Ehime 791-0295, Japan; Endoscopy Center, Ehime University Hospital, Shitsukawa, Toon, Ehime 791-0295, Japan; Department of Gastroenterology and Metabology, Ehime University Graduate School of Medicine, Shitsukawa, Toon, Ehime 790-0295, Japan

**Keywords:** ulcerative colitis, sexual dysfunction, erectile dysfunction

## Abstract

**Background:**

The association between disease activity and erectile dysfunction (ED) in patients with inflammatory bowel disease (IBD) is inconsistent, although IBD, including ulcerative colitis (UC), is reported as a risk factor for ED.

**Aim:**

The purpose of this study was to explore this association in Japanese patients with UC.

**Methods:**

In this study, we enrolled 165 Japanese male patients with UC. Information regarding the Sexual Health Inventory for Men (SHIM) score, medication, and severity of UC was obtained from medical records, self-administered questionnaires, and reports from physicians. The definition of ED and severe ED is a SHIM score <17 and <8, respectively.

**Outcomes:**

No association between severity of UC and ED was found in Japanese patients. Aging is independently positively associated with ED in patients with UC.

**Results:**

The prevalence of severe ED and ED was 47.9% and 64.9%, respectively. In this study, mucosal healing, clinical remission, duration of UC, disease extent, and medication were not associated with the prevalence of ED. Older age (≥63 years of age) was independently positively associated with ED (adjusted odds ratio, 12.93; 95% CI: 4.51-43.00) and severe ED (adjusted odds ratio, 9.02; 95% CI: 3.66-23.91).

**Clinical Implications:**

Disease severity of UC might not be associated with the prevalence of ED in patients with UC.

**Strengths and Limitations:**

This is the first study to investigate the association between several factors regarding UC activity and ED. The limitation of this study is the definition of ED based on SHIM scores.

**Conclusion:**

No association between severity of UC and ED was found in Japanese patients. As expected, aging may be independently positively associated with ED in patients with UC.

## Introduction

Ulcerative colitis (UC) is an inflammatory bowel disease (IBD). The number of patients with UC is increasing in the Asian population.^1^ In Japanese claims data for UC, the mean age of Japanese patients with UC is 41.0 years.^2^ Half of patients with UC are under 40 years, which overlaps with the sexually active period and reproductive age.

Sexual dysfunction including erectile dysfunction (ED) is in important component in quality of life.^3^ ED is a common sexual dysfunction in males. It is defined as the persistent or recurrent inability to achieve and/or maintain an erection sufficient for sexual intercourse.^4^ Vasculogenic, neurogenic, and/or hormonal dysfunction can cause ED.^5^ Atherosclerosis, central and peripheral neurodysfunction, and low testosterone are common in elderly men.^6–8^ Therefore, aging is a well-known risk factor for ED.

A meta-analysis regarding sexual dysfunction and UC showed a positive association between ED and the prevalence of IBD.^9–11^ However, the reason why UC causes ED remains unclear.

Several studies have investigated the association between the disease activity of IBD and ED. However, this association remains controversial. Disease activity is a significant risk factor for ED in patients with IBD.^9^^,^^12^ In a U.S. study, a positive relationship was found between disease activity and International Index of Erectile Function (IIEF) score in patients with UC.^13^ In a Chinese study, the prevalence of ED was significantly higher in patients with active disease than in patients in remission.^14^ In contrast, in a French study, disease activity was not associated with sexual dysfunction.^15^ After adjusting for confounding factors, no association between disease activity and ED was found in two German studies and one Dutch study of IBD,^16–18^ although disease activity was positively associated with IIEF or ED in the crude analysis. In Japanese patients with UC, no study has investigated the association between disease activity and the prevalence of ED, although an international survey indicated that the prevalence of ED is higher in the Japanese population than in other countries.^19^

In UC, intestinal inflammation may elevate inflammatory cytokines, including tumor necrosis factor α, resulting in endothelial dysfunction and atherosclerosis.^20^ Chronic inflammation may lead to vascular-related ED via elevated inflammatory cytokines, including tumor necrosis factor α.^21^ UC has also been shown to be associated with the incidence of coronary artery disease, which is closely associated with ED.^22^ Testosterone, which is associated with sexual desire, is also associated with inflammation.^23^ We speculate that the disease activity of UC might be associated with ED.

Therefore, we conducted a cross-sectional study to explore the association between the disease activity of UC and ED in Japanese patients.

## Methods

### Study design

This was a cross-sectional study using baseline data from a prospective cohort study.

### Study population

The subjects were 203 male patients with UC who visited the Department of Gastroenterology and Metabology at the Ehime University Graduate School of Medicine or several affiliated hospitals in Ehime Prefecture, Japan, as inpatients or outpatients between 2015 and 2019. All patients consented to this study and were able to answer the self-administered questionnaire. Subjects were diagnosed with UC based on endoscopic findings and met clinical, radiological, and histological criteria. After 38 patients were excluded due to missing data (endoscopic findings, 6 cases; medication, 12 cases; and Sexual Health Inventory for Men [SHIM] score, 20 cases), the final analysis sample consisted of 165 patients with UC. The protocol for this study was developed in accordance with the 1964 Declaration of Helsinki and subsequent versions of the ethical guidelines. This study was approved by the ethics committee of Ehime University of Medical Science (Approval No. 1505011). Well-trained staff obtained written informed consent from all registered patients.

### Measurements

A self-administered questionnaire elicited information regarding smoking, drinking, education, household income, diabetes mellitus, hypertension, and benign prostate hyperplasia. Current smoking was deemed positive if patients answered that they were currently smoking, regardless of the number of cigarettes. Current alcohol intake was deemed positive if patients answered that they had a drinking habit, regardless of the frequency of drinking or the amount of alcohol. Body mass index was calculated as weight (kg) divided by the square of height (m^2^). Information regarding medication for UC, disease extent, and duration of UC was obtained from medical records.

### Disease activity of UC (definition of clinical remission and mucosal healing)

The definition of clinical remission was based on no rectal bleeding and no abnormally high stool frequency (cutoff frequency: 3 times per day). Mucosal healing (MH) was defined as Mayo Endoscopic Score (MES) category 0.^24^ A certified endoscopist evaluated endoscopic activity by total colonoscopy and reported endoscopic findings and key images. A single endoscope specialist was responsible for evaluating MES and MH close to the time that patients completed the questionnaire and was blinded to other findings, including other characteristics.

### Assessment of ED

The SHIM is a validated, abridged, 5-item version of the 15-item IIEF questionnaire.^25^ In the present study, we used the following outcomes: (1) ED was defined as present when a subject had a SHIM score <17; and (2) severe ED was defined as present when a subject had a SHIM score <8.

### Statistical analysis

Age was divided into 3 categories based on the distribution of subjects in this analysis: (1) young age, 17 to 42 years old (reference); (2) middle age, 43 to 62 years old; and (3) old age, ≥63 years old. Duration of UC, medication for UC, and disease extent were each divided into 2 categories, as follows: long duration (<7 or ≥7 years,), number of drugs for UC (<2 or ≥2), and disease extent (pancolitis or nonpancolitis). Crude odds ratios (ORs) and their 95% CIs for severe ED and ED in relation to each of the factors were estimated using logistic regression analysis. Multiple logistic regression analyses were used to adjust for potential confounding factors. Age, current drinking, current smoking, body mass index, MH, clinical remission, long duration of UC, number of drugs for UC, and disease extent were selected a priori as potential confounding factors. Trend of an association was assessed using a logistic regression model assigning consecutive integers to the categories of the age variables. All statistical analyses were performed using SAS software package version 9.4 (SAS Institute). All probability values for statistical tests were 2-tailed, and *P* < .05 was considered statistically significant.

## Results

### Patient characteristics

**Figure 1:**
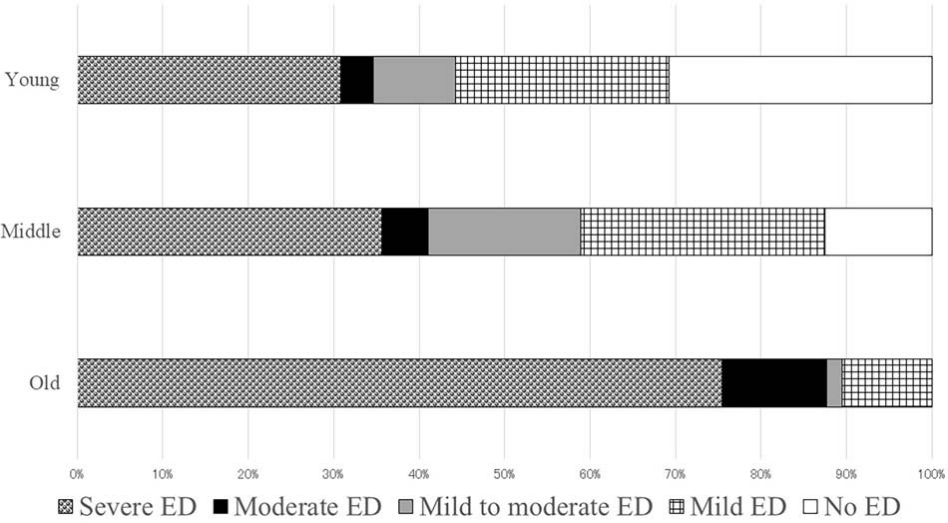
Severity of erectile dysfunction (ED) and old age. In old age, no patients with ED were found.

Characteristics of the 165 patients with UC are shown in Table 1. The average age, duration of UC, and body mass index were 52.1 ± 16.9 years, 7.7 ± 8.1 years, and 22.23 ± 4.45 kg/m^2^, respectively. The percentages of low, medium, and high education and low, medium, and high household income were 53.9%, 13.9%, 32.1%, 21.1%, 46.1%, and 32.7%, respectively. The percentages of MES 0, MES 1, MES 2, MES 3, MH, and clinical remission were 24.2%, 32.7%, 35.8%, 7.3%, 24.2%, and 57.6%, respectively. The prevalence of mild ED, mild-to-moderate ED, moderate ED, severe ED, and ED were 21.1%, 9.7%, 7.3%, 47.9%, and 64.9%, respectively. In old age, no patients with ED were found (Figure 1).

**Table 1:** Clinical characteristics of 165 study participants.

Age, y	52.1 ± 16.9
17-42 y, %	52 (31.5)
43-63 y, %	56 (33.9)
≥63 y, %	57 (34.6)
Education	
Low (≤ 12 y)	89 (53.9)
Middle (12-16 y)	23 (13.9)
High (≥16 y)	53 (32.1)
Household income	
Low income (≤¥2 999 999/y)	35 (21.1)
Middle income (¥3 000 000-¥7 999 999/y)	76 (46.1)
High income (≥¥8 000 000/y)	54 (32.7)
Diabetes mellitus, %	12 (7.3)
Hypertension, %	35 (21.2)
Benign prostate hyperplasia, %	13 (7.9)
Disease extent, pancolitis/left-sided/proctitis/other^a^	75/41/44/5
Duration of UC, y	7.7 ± 8.1
BMI, kg/m^2^	22.23 ± 4.45
Current smoking, %	16 (9.7)
Current drinking, %	77 (46.7)
Medication	
5-aminosalicylates, %	155 (93.9)
Prednisolone	35 (21.2)
Thiopurines, %	24 (14.6)
TNF-α monoclonal antibody, %	6 (3.6)
MES	1.26 ± 0.91
0, %	40 (24.2)
1, %	54 (32.7)
2, %	59 (35.8)
3, %	12 (7.3)
Complete mucosal healing (MES <1)	40 (24.2)
Clinical remission	95 (57.6)
SHIM score	11.0 ± 8.3
Mild ED (17 ≤ SHIM score < 22), %	35 (21.2)
Mild-to-moderate ED (12 ≤ SHIM score < 17), %	16 (9.7)
Moderate ED (8 ≤ SHIM score < 12), %	12 (7.3)
Severe ED (SHIM score <8), %	79 (47.9)
ED (SHIM score <17), %	107 (64.9)

Values are mean ± SD or n (%).

Abbreviations: BMI, body mass index; ED, erectile dysfunction; MES, Mayo Endoscopic Score; SHIM, Sexual Health Inventory for Men; TNF, tumor necrosis factor; UC, ulcerative colitis.

^a^Right-sided, segmental colitis, and postoperative patients (lack of any preoperative medical records for postoperative patients)

### Association between severity of UC and ED

Table 2 shows the association between severity of UC and ED in this cohort. MH, clinical remission, long duration of UC, number of drugs for UC, and disease extent were not associated with ED. In crude analysis, older age was positively associated with ED (crude OR, 10.72; 95% CI, 4.14-31.89). After adjustment, old age was independently positively associated with ED (OR, 12.93; 95% CI, 4.51-43.00; *P* for trend = .001).

**Table 2:** Crude and adjusted ORs and 95% CIs for the associations between disease activity of UC and ED.

Variable	Prevalence (%)	Crude OR (95% CI)	Adjusted OR (95% CI)
Severe to moderate ED			
MH (MES <1)			
No	68/125 (54.4)	1.00	1.00
Yes	23/40 (57.5)	1.13 (0.56-2.35)	0.98 (0.37-2.52)
Clinical remission			
No	38/70 (54.3)	1.00	1.00
Yes	53/95 (55.8)	1.06 (0.57-1.98)	0.86 (0.38-1.94)
Long duration of UC			
<7 y	52/94 (55.3)	1.00	1.00
≥7 y	39/71 (54.9)	0.98 (0.53-1.83)	0.85 (0.39-1.85)
Number of drugs for UC			
<2	56/106 (53.3)	1.00	1.00
≥2	35/60 (58.3)	1.23 (0.65-2.34)	1.47 (0.68-3.23)
Pancolitis			
No	50/90 (55.6)	1.00	1.00
Yes	41/75 (44.4)	0.97 (0.52-1.79)	1.32 (0.68-3.23)
Age			
Young	18/52 (34.6)	1.00	1.00
Middle age	23/56 (41.1)	1.32 (0.60-2.90)	1.77 (0.74-4.39)
Old age	50/57 (87.7)	13.49 (5.34-38.31)	18.28 (6.57-58.26)
*P* for trend			.001

Values are n/n (%), unless otherwise indicated. ORs were adjusted for age, body mass index, current drinking, current smoking, disease duration, number of drugs for UC, disease extent, clinical remission, and MH.

Abbreviations: ED, erectile dysfunction; MES, Mayo Endoscopic Score; MH, mucosal healing; OR, odds ratio; UC, ulcerative colitis.

### Association between severity of UC and severe ED

Associations between severity of UC and severe ED are shown in Table 3. Old age was independently positively associated with severe ED (adjusted OR, 9.02; 95% CI, 3.66-23.91; *P* for trend = .001). No association between other factors regarding UC and severe ED was found.

**Table 3:** Crude and adjusted ORs and 95% CIs for the associations between disease activity of UC and severe ED.

Variable	Prevalence (%)	Crude OR (95% CI)	Adjusted OR (95% CI)
Severe ED			
MH (MES <1)			
No	57/125 (45.6)	1.00	1.00
Yes	22/40 (55.0)	1.46 (0.71-3.01)	1.77 (0.72-4.45)
Clinical remission			
No	34/70 (48.6)	1.00	1.00
Yes	45/95 (47.4)	0.95 (0.51-1.77)	0.66 (0.30-1.44)
Long duration of UC			
<7 y	46/94 (48.9)	1.00	1.00
≥7 y	33/71 (46.5)	0.91 (0.49-1.68)	0.72 (0.34-1.50)
Number of drugs for UC			
<2	48/105 (45.7)	1.00	1.00
≥2	31/60 (51.7)	1.27 (0.67-2.40)	1.58 (0.75-3.37)
Pancolitis			
No	44/90 (48.9)	1.00	1.00
Yes	35/75 (46.7)	0.92 (0.49-1.69)	1.18 (0.58-2.43)
Aging			
Young	16/52 (30.8)	1.00	1.00
Middle age	20/56 (35.7)	1.25 (0.56-2.82)	1.72 (0.71-4.28)
Old age	43/57 (75.4)	6.91 (3.05-16.55)	9.02 (3.66-23.91)
*P* for trend			.001

ORs were adjusted for age, body mass index, current drinking, current smoking, disease duration, number of drugs for UC, disease extent, clinical remission, and MH.

Abbreviations: ED, erectile dysfunction; MES, Mayo Endoscopic Score; MH, mucosal healing; OR, odds ratio; UC, ulcerative colitis.

## Discussion

In this study, aging was independently positively associated with the prevalence of ED in patients with UC, whereas the severity of UC was not associated with the prevalence of ED. This is the first study to investigate the association between disease severity of UC and ED in Japanese patients with UC.

ED is a common sexual dysfunction in males. In the Massachusetts Male Aging Study, the prevalence of ED in the forties is approximately 40% and increases 10% per decade.^7^ ED is defined as the persistent or recurrent inability to achieve and/or maintain an erection sufficient for sexual intercourse.^8^ Several studies have identified a positive association between IBD (including UC) and ED. The prevalence of IBD was positively associated with ED in a meta-analysis.^9^ In a nationwide cohort from Denmark, ED medications were more frequently used in patients with IBD than men without IBD.^10^ In a Taiwanese cohort study of 1845 patients with IBD, IBD was independently positively associated with ED.^11^ Endothelial dysfunction and atherosclerosis might cause ED. Surrogate markers for atherosclerosis, including pulse wave velocity and carotid intima-media thickness, were higher in patients with IBD than those of control individuals.^26^ Additionally, a positive association between duration of IBD and pulse wave velocity was found in a Croatian study.^27^ Chronic inflammation due to UC might be associated with endothelial dysfunction and atherosclerosis, resulting in ED. However, the reason underlying the association between UC and ED remains unclear.

As mentioned previously, some studies have shown an association between the disease activity of IBD and sexual dysfunction including ED. In a U.S. study of 69 patients with IBD, including 28 patients with UC, a positive relationship between disease activity and IIEF was found in patients with IBD.^13^ In a Chinese study of 208 patients with IBD including UC, the prevalence of ED was significantly higher in patients with active disease compared with patients in remission.^14^ In a French study of 358 patients with IBD, disease activity was not associated with sexual dysfunction.^15^ In 2 German studies and 1 Dutch study of males with IBD, disease activity was associated with ED in the crude analysis, but no association between disease activity and IIEF was found after adjustment.^16^^,^^17^ In a Spanish study of 355 patients with IBD and a New Zealand study of 159 patients with IBD, disease activity was associated with sexual dysfunction only in female patients.^28^^,^^29^ The discrepancies between findings in this study and those of previous studies might be explained, at least in part, by sample size, distribution of age, rate of previous surgery, and the definition of ED. However, sample sizes, including that of the present study, might be too small to confirm the association between disease activity and ED in patients with IBD. In this study, most patients with UC had received treatment to control inflammation for a long time. Thus, treatment for UC might mask the association between disease activity and ED.

Aging is a well-known risk factor for ED. Aging is strongly associated with atherosclerosis. Age-related decline in serum testosterone is also observed.^8^ Penile erection is a vascular phenomenon resulting from smooth muscle relaxation, arterial dilatation, and venous restriction. The effects of testosterone on libido and sexual behavior are well established.^5^ In a U.S. population-based study, age was the variable most strongly associated with ED.^7^ In the Japanese population, aging is positively associated with ED.^30–34^ In a Taiwanese cohort study of 1845 patients with IBD, age (≥64 years) was independently positively associated with the incidence of ED.^11^ Thus, the association between aging and ED found in the present study was consistent with previous studies.

Conclusive evidence regarding the association between UC and ED in Japanese patients is lacking. The number of patients with UC is still increasing in Japan, and the prevalence of ED was higher in the Japanese population than in other countries in an international survey.^23^^,^^30^ However, only one Japanese study of 61 patients with UC (30 males and 31 females) after ileal pouch–anal anastomosis showed low sexual activity in patients with UC.^35^ Further research regarding sexual dysfunction and ED in Japan is warranted.

Our survey has several limitations. First, the cross-sectional study design does not allow the establishment of a causal relationship between aging and ED. Second, in this study, we utilized a self-administered questionnaire. Although there are no data on the validity of ED severity, any possible misclassification of nondiscriminatory exposure would introduce bias toward the null hypothesis. Third, the sample size of this study was too small to confirm disease activity and ED; larger studies are needed to confirm this association. Finally, selection bias may have influenced the results of this study. The percentage use of biologic drugs might be low in this cohort, and this study population was likely not representative of all Japanese patients with UC. However, the mean age, sex ratio, and drug dosage in this study were similar to those in Japanese national surveys of UC.^2^

## Conclusion

The severity of UC might not be associated with ED in Japanese men with UC, whereas aging was independently associated with ED. Healthcare professionals should be aware that EDs are common in UC and increase with age, regardless of UC activity.

## Funding

This research did not receive any specific grant from funding agencies in the public, commercial, or not-for-profit sectors.

*Conflicts of Interest*: The authors declare that they have no conflicts of interest.

## Data Availability

The datasets used and/or analyzed during the current study are available from the corresponding author on reasonable request.
